# The Public Spirit of Property Owners

**Published:** 1918-06-01

**Authors:** 


### THE PUBLIC SPIRIT OF PROPERTY OWNERS.

Some years ago in a large provincial town it was proposed to establish a tuberculosis dispensary; and the proposal being carried, the primary question of a site came up. It was, however, found to be a very thorny question, for each suggestion met with the strongest opposition from residents in the locality concerned. Education and presumed enlightenment or sympathy proved to be no passport at all to an understanding being reached in the matter: quite a dead-set was made against the harassed committee of management, by landlords and tenants both, in the medical quarter of the town, when it was endeavoured to rent premises there. Ministers of religion, both Established and Nonconformists, fought as stoutly as the rest against such new neighbours. Eventually, to avoid further waste of valuable time, two very ill-adapted rooms in the municipal Public Health Offices were cleared out, and the patients crowded in.

The psychological phenomena deducible from such cases—for instance, the easy accessibility of primitive instincts, in spite of a veneer of training and education—are highly interesting. Nevertheless, it is rather the practical aspect that concerns us here, and the practical aspect is visible in a judgment just delivered by Mr. Justice Eve in an action brought by freeholders and leaseholders in the neighbourhood of Cardigan House, Newport, Monmouthshire, to restrain the King Edward VII. Welsh National Memorial Association for Prevention of Tuberculosis from carrying on that establishment as a hospital for surgical tuberculosis. The heads of the judgment give the events so often familiar to promulgators of new hospitals. As soon as the free offer of Cardigan House for the purpose stated was known, "strong opposition to its being given effect to was raised by some of the neighbouring owners and residents," the grounds being advanced that the institution would be a source of danger and a nuisance to the neighbourhood. These efforts failed, and less than a year ago the institution was opened. Now they have been renewed. The judicial ruling is that the efforts of the plaintiffs to secure removal of the hospital altogether by proving it a nuisance, either intrinsically or by its carrying on, had failed. The evidence was judged overwhelming that all treatment was carried out with skill and care and under the most approved modern conditions. The sights might now and then be distressing and even annoying, but the only real and substantial objection could be the danger of infection, and that was negatived by weighty medical evidence. So far the action had failed, said Mr. Justice Eve. It appeared, however, that a legal provision did exist forbidding the premises to be used otherwise than as a dwelling-house, of which provision their present employment obviously involved a breach. An injunction was therefore granted, but in view of the overcrowded condition at present of all hospitals, whereby the closing of Cardigan House would involve the return of its patients to their own homes, with consequent damage to their health by surroundings often most unfavourable, the operation of the injunction was suspended for six months. Also the defendants were given liberty to apply for a further suspension should the present conditions still obtain at the end of that period.

Perhaps, like Mr. Justice Eve, we have already let our own opinion sufficiently appear as to the equity of this matter. *Per contra*, it can be said that it is well sometimes to look a gift horse in the mouth, and that presumably a day or two's employment of a good solicitor in the first place might have revealed the legal clause upon which the issue as above stated turned, of which the generous donor was no doubt ignorant, and saved much ado and much expense, and probably all the upset of a removal. For there is a possibility, of course, that although, as we all hope, plaintiffs and defendants and onlookers alike, "present conditions" will not outlast six months, yet this injunction may. The war has fostered tuberculosis into gianthood. The war threatens this country, as it does others, with bankruptcy, to avert which, if for no other reason, the public health must be saved. "God Save the People," sang the rhymer, and more than rhymer, Elliott. How does "The neighbourhood objects to a hospital" sound against that?

